# Priorities for modelling arbovirus transmission under climate change

**DOI:** 10.1016/j.molmed.2025.05.010

**Published:** 2025-06-17

**Authors:** Ilaria Dorigatti, Katy A.M. Gaythorpe, Victoria M. Cox, Francis A. Windram, Lauren Cator

**Affiliations:** 1 MRC Centre for Global Infectious Disease Analysis, School of Public Health, Imperial College London, London, UK; 2 Department of Life Sciences, Imperial College London, London, UK

## Abstract

The transmission potential of arboviruses is extremely sensitive to environmental conditions. This sensitivity is due to both their intimate relationship with ectothermic vectors and, in many cases, also to the involvement of multiple host species in zoonotic transmission cycles. Here, we review how climate change will alter the transmission ecology and risk of these important infections. The challenge of predicting how climate change will impact these systems is daunting, but the need for tools to manage arbovirus risk under climate change is urgent and imperative. We argue that the development of climate-driven mechanistic models of disease transmission informed by empirical surveillance data is urgently needed to inform future responses and for generating the evidence that policy needs to tackle this global public health risk.

## Arboviruses in a changing climate

Arbovirus is a blend of the words ‘arthropod-borne’ and ‘virus’ and denotes viruses transmitted by a diverse range of organisms including mosquitoes, midges, ticks, and sandflies. These diseases are highly sensitive to environmental changes in part because these organisms are ectothermic (see Glossary). Climate change combined with deforestation, anthropogenic land-use and urbanization have increased interactions between previously isolated human and animal populations [1]. The increasing size of the population exposed to disease due to both rising urbanisation and population density along with warmer conditions in many areas has set the stage for the emergence and re-emergence of new pathogens of pandemic potential, including arboviruses [2–6]. In this article, we summarise the current understanding, knowledge gaps, and uncertainties of how climate affects arbovirus risk now and how it will affect it in the future. Using an evidence-based data-driven approach to understand the mechanisms underlying this relationship is vital for developing models and tools to project into the future the likely patterns of arbovirus transmission. These models and tools can provide critical insights to local and global policy makers as they can inform programmatic decisions on disease surveillance, outbreak preparedness, response, and intervention planning as well as climate policy.

## Current understanding of the effect of climate change on arbovirus transmission risk

**Figure 1:**
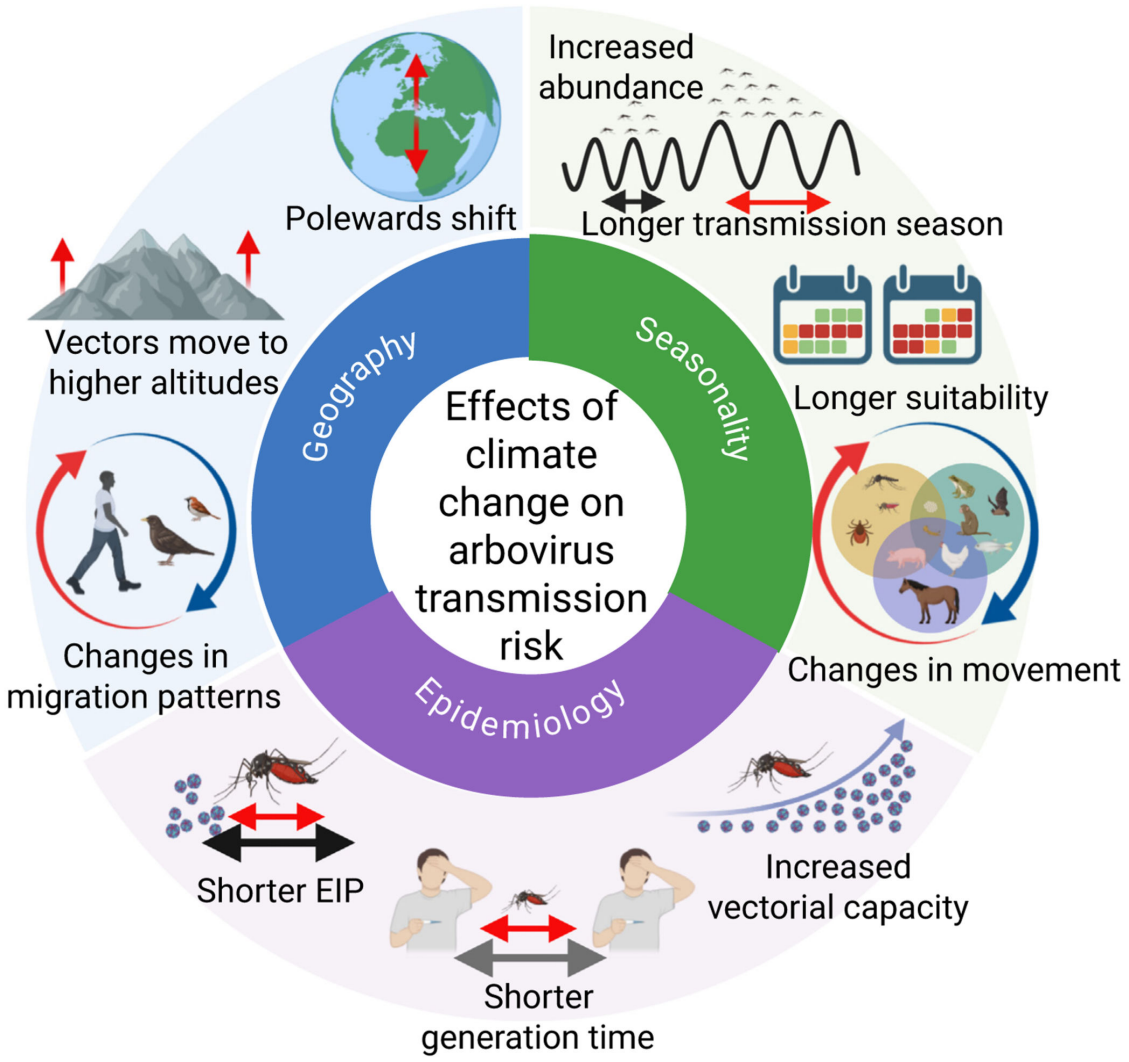
Expected effects of climate change on the epidemiology, geographical and spatial spread of arboviruses, and their seasonality. Abbreviation: EIP, extrinsic incubation period.

Climate change is expected to affect arbovirus transmission in three ways: (i) by expanding the geographic range and suitability of arbovirus vectors and hence the occurrence of these diseases into new areas, (ii) by altering the dynamics, including the timing and duration of the transmission season, and (iii) by modifying the intensity of transmission and the size of future outbreaks in both epidemic and endemic settings through multiple mechanisms summarised in Figure 1 (Key figure) and discussed in detail below.

## Key figure

Graphical summary of the expected effects of climate change on arbovirus transmission

Warming temperatures are expected to favour the establishment of vectors in temperate regions that are currently deemed largely unsuitable. This includes expansion pole-wards and to higher altitudes, resulting in an increase in disease transmission in many regions under climate change scenarios [4,7–13]. Some of this expansion is expected to be driven by changes in the migratory patterns of the animal reservoirs that sustain the zoonotic transmission upon which some arboviruses, such as West Nile virus, depend. Changes in temperature [14–17], habitat fragmentation, human-mediated changes in land-use [16], vegetation [17] and agricultural practises, such as irrigation, can lead to unexpected vector and host movement, biodiversity loss [18], and virus dispersal [16,19–21]. The geographic expansion of vectors will place new populations at risk of infection, and these populations will either have no immunity or different immunity profiles compared to the populations where arboviruses currently circulate. For example, changes in climate suitability for *Aedes* mosquitoes is predicted to have increased the population at risk between the 1980s and 2020 by around two billion people [22]. A further increase of more than two billion people is estimated from 2015 to 2080 assuming the 2.0 shared socioeconomic pathway (SSP) scenario [23]. Climate change will also likely result in conditions that are either too hot or too dry for vectors to survive, thus potentially resulting in reductions in vector populations and disease risk at the local scale in specific locations which currently experience a high arbovirus burden [5,9,24].

Climate change is also expected to modify the seasonality, or phenology, of the occurrence of vectors which, in turn, is expected to affect the timing and duration of the local transmission seasons. This is particularly true for cycles involving multiple host species or those that require the interaction of overlapping stages of vectors. For example, co-feeding between larval and nymphal ticks is an important part of the dynamics of tick-borne encephalitis (TBE), and phenological shifts in the abundance of ticks and nymph life stages can have major impacts on transmission [4]. Seasonal migration patterns of animal reservoirs have been shown to be hugely influential in virus transportation both for West Nile virus [15] and for tick-borne diseases such as Crimean–Congo haemorrhagic fever [25].

Extreme weather events will be more frequent with climate change. Events such as drought and floods can affect movement of livestock and wildlife, leading to overlap with vector breeding sites and providing blood-meal availability which can increase the transmission risk to humans [15,17,26–31]. Furthermore, drought – beyond altering water-storage practices – can reduce the population of vector predators (such as frogs) thus increasing the abundance of vectors [32] and, in turn, increased vector abundance can affect the risk that these diseases pose to human and animal populations. Notably, for immunizing diseases that circulate endemically, vector abundance is not necessarily a direct and representative measure of population risk, as immunity modulates the individual- and population-level risk of infection. However, in settings where there is no pre-existing immunity, vector population dynamics can be among the key drivers of arbovirus risk [33]. Experimental studies suggest that climate dependencies modulate virus–vector interactions and the risk of infection of vectors [34], and that warmer conditions and reduced variations in daily temperatures can lead to increased vectorial capacity [34] and vector competence [35]. In Box 1, we discuss the expected effect of temperature dependencies on the generation time [2] – and specifically shorter average generation times under warming temperatures – and on the expected increase in the transmission potential of several flaviviruses transmitted by *Aedes aegypti* [36] on outbreaks dynamics.

From an intervention perspective, heat stress [37] and prolonged heatwaves [38] could adversely affect the efficacy of *Wolbachia* as a biological form of *Ae. aegypti* control [39,40]. Therefore, the locations most affected by these changes in the climate could also see larger reductions in the efficacy of *Wolbachia*. However, whilst experimental studies show that the density of *Wolbachia* in naturally occurring *Aedes albopictus* [41] populations may not be negatively affected by warmer climate conditions [42], modelling projections suggest that climate change could accelerate *Ae. albopictus* invasion into new areas [43].

## Current understanding of how climate influences vector biology

A trait is any measurable characteristic of an organism. Many vector traits, such as the development rate, biting rate, host preference and mortality, have important effects on arbovirus dynamics [44–46]. One of the best-characterised dependencies is the strong non-linear relationship between traits and temperature, which shows that vectors – and in particular mosquitoes – develop faster, bite more frequently, and live longer at certain optimal temperatures. This relationship between trait performance and climate conditions can be captured by thermal performance curves (TPCs) (Box 2) which, together with thermal tolerance experiments, provide evidence of the geographic limits and distribution of species [46–48]. Whilst published TPCs have been typically generated from experiments on mosquitoes and in laboratory-derived colonies [49], which do not necessarily characterise the variation observed in naturally occurring populations, these observed relationships provide a strong biological and mathematical foundation to explain current vector population distributions and abundances. Notably, thermal dependencies not only affect the life cycle of the vector but also determine the way that these organisms interact with viruses. Once vectors become infected by feeding on an infectious host, the virus must replicate – and in many cases disseminate – within the vector before the vector can transmit the virus onwards to a new host, a period which is termed the extrinsic incubation period (EIP). The EIP can range from a few days to weeks depending on the pathogen, vector, and the temperature at which viral replication occurs [50–52]. Warmer temperatures have been shown to increase the vectorial capacity of mosquitoes for different arboviruses in a virus-specific manner [53]. Beyond temperature, humidity has been suggested to play a key role along with temperature on vector traits and viral transmission [54], but experimental data reflecting how temperature and humidity patterns affect the biology and transmission potential of the main arbovirus vectors, including *Ae. aegypti* and *Ae. albopictus*, are currently lacking.

## Climate driven modelling frameworks: strengths, weaknesses, and current gaps

Quantitative estimates of the potential impact of climate change on arbovirus transmission have been generated using different metrics of disease transmission risk. These typically consist of (i) distribution maps that describe or predict the presence and absence of vectors [55–57], and viruses [58,59] in specific locations or regions, and (ii) suitability indices, which map the suitability of the local environment to virus transmission, for example through temperature suitability [60,61], index P [62], vectorial capacity [63], the probability of disease introduction and establishment [64] and the basic reproduction number (R_0_) [53,65]. The R_0_ is a fundamental measure of transmission potential of arboviruses (and pathogens in general) [66] quantifying the average number of secondary infections generated by a typical infection in a fully susceptible population at the start of an epidemic, and the relatively high estimates obtained for arboviruses [66] imply that outbreaks are typically explosive and difficult to control (Box 1). To date, a substantial body of research has focussed on including temperature into models of R_0_ for arboviruses using a variety of approaches, including non-linear relationships between vector traits and temperature [49,67,68]. These trait-based R_0_ models, derived from extensions of the classic Ross–McDonald model [69,70], have been used to project forward in time the effect of warming temperatures under different representative concentration pathway (RCP) scenarios [71]. These approaches require empirical data on how traits vary at small intervals across a wide range of temperatures, but due to experimental costs and resources, observational data are typically generated over a limited number of individual temperature points and inference on the temperature dependence beyond the observed points is made using chosen functions and calibration methods. One common assumption made by trait-based R_0_ models is the absence of intraspecific variation in trait temperature dependence, and that the vector and virus will not adapt to climate change, despite published data suggesting that evolutionary adaptation has occurred in both [72–74].

Notably, the trait-based R_0_ models that have been used to investigate the likely impact of climate change only assume temperature dependencies, and to date, a validation of how these R_0_ estimates capture the epidemiology of the disease, such as the typical age at which infections occur, is lacking. Catalytic models are used to estimate the force of infection (FOI) from the observed age-distribution of seroprevalence or case-notification data [75–78]. Machine-learning models using environmental, climate, and demographic data have been used to reconstruct and project spatial heterogeneities in FOI and R_0_, including in locations with no surveillance data. For dengue, global and regional FOI maps have been particularly useful to assess the potential impact of interventions [79,80], despite the fact that these maps are static and do not project how the transmission intensity may vary under climate change.

In terms of modelling frameworks, statistical approaches, including machine learning, typically quantify the association between observational data, for instance response variables such as vector abundance [81] or case incidence [24], and climatic, environmental, demographic or socioeconomic predictors. These approaches then use the estimated relationships and effect sizes to extrapolate the effect of climate change scenarios. Whilst the phenomenological nature of this approach is typically successful at reconstructing the highly variable nature of arbovirus transmission with good precision, regression-based models typically do not account for (i) demographic changes in population structure (e.g., shifts towards older population), (ii) human population immunity (which is a major driver of the outbreak risk and burden of infection and disease), (iii) changes in surveillance and disease reporting (e.g., changes driven by the availability of new diagnostics), and (iv) the potential impact of interventions. Furthermore, regression-based models do not always account for non-linear relationships and thus cannot capture the fundamental biological drivers behind the observations.

By contrast, mechanistic models include the processes through which the number of infections in a population, and hence the risk of infection, change with time. These models describe the way infections are generated in a population during an epidemic through a feedback loop determined by the contact patterns between vectors and humans and, in turn, can be designed to explicitly capture the sensitivity of disease surveillance. As such, these models provide the unique advantage of projecting the likely impact of interventions, including those reducing transmission (e.g., protecting against infection) and disease (e.g., reducing the probability of developing symptoms) as observed from clinical trials onto the general population, even when these interventions have not been trialled *in situ*.

Climate-driven mechanistic transmission models that build on TPCs have been proposed as theoretical or simulation-based frameworks [82], and to date a few models have been used to capture summary characteristics of epidemics [83]. However, reconstructing the observed disease dynamics through the calibration of climate-driven compartmental models to time series data remains an open challenge, especially for dengue, due to the complex immunity profile of endemic populations, the limited number of seroprevalence studies, and inferential and computational challenges associated with reconstructing the immunity profile of the population.

## Opportunities for climate-driven modelling of arboviruses

The direct and indirect effects of climate and environmental changes on human and animal populations, and the complex nature of arbovirus transmission encompassing feedback loops between populations, require an improved understanding of how climate drives disease transmission from a mechanistic perspective. The development of mechanistic climate-driven disease models, and the implementation of computationally-efficient methods to calibrate these models to observed surveillance data, will allow the inference of unobserved processes and the generation of refined projection of the expected impact of climate change scenarios on arbovirus infection and disease burden.

To achieve this, it is necessary to generate entomological and epidemiological data from a wide range of geographies where there is evidence of arbovirus transmission but insufficient data to reconstruct the past and current risk, which is the premise to project the likely effect of climate change into the future. For arboviruses circulating within animal reservoirs, the monitoring of changes in the migration and mobility patterns of the animal reservoir, such as migratory birds and non-human primates, is a priority [14,84] to learn how to identify changing patterns in at-risk regions [85].

Another current priority is characterising the extent of the within-species variations in TPCs observed in field-derived mosquito populations as compared to the average TPCs derived from laboratory-adapted populations, and in turn, assessing its effect on the R_0_ and on the dynamics and burden arbovirus infection in both epidemic and endemic settings. Due to limitations in infrastructure and resources, information on the thermal performance and tolerance of vectors across endemic regions is currently lacking. Investing in field and laboratory capacity to generate TPCs – and more generally entomological data – in the settings that are currently most affected by arboviruses is an urgent priority both for modelling and surveillance purposes. These data will allow validation of current estimates and underlying assumptions, for instance estimation of how field variation in the thermal performance of vectors across locations affects the observed transmission dynamics from human disease surveillance data or other epidemiological data generated from seroprevalence studies.

From an experimental perspective, fully characterising the TPCs of traits, including at the cooler and hotter end of curves, is vital to characterise the current distribution boundaries and inform thermal limits which are otherwise inferred from theoretical models (e.g., Brière or quadratic functions). Better understanding of thermal adaptation, and how thermal limits may shift, is another key priority as TPCs are often assumed to be static but there is experimental evidence that mosquitoes exposed to heat stress quickly evolve to accommodate increasing temperatures within a few generations [72].

Furthermore, whilst temperature is a critically important variable, greater consideration of the interaction between temperature and humidity [54] and the effect that precipitation has on the carrying capacity is needed to integrate indirect impacts of climate on vector distribution, abundance, and shifts in host availability and habitat. Further accounting for how local hydrological and infrastructural conditions affect the extent to which temperature, humidity, and precipitation modify the carrying capacity will further improve our ability to anticipate disease risk in a changing climate [4].

The extent to which it will be possible to infer variations in plasticity and transfer observed effects across locations is unclear, and more research is needed to develop a theoretical understanding that can be used to project forward in time the effect of vector and vector–virus evolution.

Beyond the generation of disease surveillance data and new empirical evidence of how the climate affects the carrying capacity and within-species trait dependencies, the development of refined models of disease transmission embedding entomologically mediated climate relationships (including thermal evolutionary adaptation), combined with the implementation of computationally efficient inferential frameworks, is needed. Including a representation of the demographic structure of populations and their shifts [86,87] will be crucial to project changes in the epidemiology, clinical presentation (see Clinician’s corner) and healthcare demand posed by arboviral diseases locally and globally. Further developing these climate-driven mechanistic models to account for structural changes to the urban landscape during and after the construction phase (which is often reported as being a major source of infections in expanding cities), changing health infrastructures, anthropogenic activity, as well as changes in mobility and migration patterns, would further refine our collective understanding of the future of arbovirus transmission under a changing climate. These models could shed light on the historical impact of anthropogenic climate change on arbovirus transmission and help to disentangle the effect of changes in the climate from changes in urbanization, sanitation, land use, mobility, demographic and socioeconomic conditions, which is an open question.

Finally, the development of efficient software and user-friendly tools to simulate (and ideally calibrate) arbovirus transmission dynamics using climate-driven models will catalyse the use and operationalisation of these models in the regions most affected by arboviruses for surveillance, health system, and intervention planning – and potentially for urban landscape planning too – for the health, societal and economic benefit of our and future generations.

## Concluding remarks

Climate change will impact arbovirus transmission through multiple mechanisms and impact the range, seasonality, and magnitude of future epidemics, thus resulting in a changing infection and disease burden in both endemic and currently unaffected areas. There are several uncertainties and unknowns around current and future drivers of population and disease transmission dynamics, disease burden, and the potential impact of interventions under a changing climate (see Outstanding questions). Projecting these changes is subject to uncertainty but the development of climate-driven mechanistic models is a first step to synthesise the existing evidence, propagate the uncertainty and develop tools to improve the currently available estimates of arbovirus risk under climatic change. These insights will be vital for informing adequate surveillance activities in endemic and at-risk regions and for planning the implementation of intervention strategies, climate policy and resource distribution. All models have uncertainties due to their abstract nature and simplifying assumptions, but they can only be as good as the data that informs them. Despite current uncertainties in global health research and funding [88], key global priorities for outbreak surveillance and response continue to include strengthening entomological, zoonotic and human surveillance, monitoring ecological and epidemiological changes in the distribution, seasonality, and abundance of vector populations and the diseases that they transmit, and effective global communications. Filling key knowledge gaps on the climate dependency and adaptation of vector populations across different transmission settings now will better prepare us for responding to health emergencies in the future.

## Highlights

The transmission of arboviruses such as dengue, West Nile virus, Crimean–Congo haemorrhagic fever and yellow fever is increasing in intensity and distribution.

There is consensus that climate change is driving and will continue to play a key role in the observed increase in the risk of arbovirus transmission. However, there are gaps in our understanding of the relative importance of the different factors that contribute to this risk.

In addition to the development of new modelling frameworks, we must improve our understanding of how vectors, viruses, and the ecology of host–virus interactions will be impacted by climate change in the context of shifts in land use and biodiversity.

Developing a mechanistic understanding of how climate affects transmission is a priority to assess the potential impact of current and new interventions in the future.

## Glossary

Basic reproduction number (R_0_): also called ‘R naught’, this is an epidemiological parameter which gives the expected number of secondary infections (or offsprings) generated by a typical infection in a fully susceptible population at the start of an epidemic
Carrying capacity: the maximum population size that an environment can sustain due to resource limitations. In mathematical terms, the carrying capacity is a key parameter of the Verhulst model which generates the logistic growth model that is used in ecology, epidemiology and other fields
Ectothermic: cold-blooded, it refers to organisms which cannot regulate their body temperature and whose internal thermal state is dependent exclusively on the temperature of the external environment
Extrinsic incubation period (EIP): the time between exposure of a vector to an arbovirus through the uptake of an infectious blood meal to when the vector becomes infectious, that is, able to transmit the virus onwards when biting a host.
Force of infection (FOI): the rate at which susceptible individuals are infected. It is typically expressed in years, but the unit of measure can be different. Historically, catalytic models applied to seroprevalence data assume that the FOI is constant-in-time in endemic settings and the current global dengue FOI map does not incorporate temporal variations.
Generation time: the time between infection of a primary case and infection of a secondary case. This is often described by a probability distribution characterising the probability of the time difference between infection of the infector and infection of the infectee.
Representative concentration pathway (RCP): climate change scenario describing future greenhouse gas concentrations up to 2100. The different RCPs can be translated into levels of temperature change which are often used to project changes in arbovirus risk in the future
Shared socioeconomic pathways (SSP): narrative, linked to the RCPs, which describe the socioeconomic trends underlying the scenarios and hence five different ways in which the world might evolve. The basic elements of the SSPs at the country-level include GDP, population, education, and urbanization. Integrated assessment models (IAMs) provide further region-specific outputs.
Vector competence: the ability of a vector to transmit a pathogen. It is typically determined through experimental infection studies which assess the ability of a vector to transmit the virus after the ingestion of viral particles through an infectious blood meal.
Vectorial capacity: the transmission potential of a vector. In mathematical terms, the simplest formulation of vectorial capacity is expressed as a function of the vector density (m), the biting rate (a), the vector competence (b), the daily survival probability (p) and the extrinsic incubation period (EIP) through the formula $VC=\frac{\text{m}\;{\text{a}}^{2}\text{b}\;{\text{p}}^{EIP}}{-\text{lnp}}.$ More complex models have been proposed in the literature for arboviruses.

## Box 1 R_0_, generation time, and epidemic dynamics

The basic reproduction number (R_0_) and the generation time are two key epidemiological parameters that respectively determine the size and speed, or temporal dynamics, of an epidemic. Epidemics with the same R_0_ but shorter generation times generate more explosive outbreaks. Similarly, larger R_0_ values under the same generation time give rise to larger epidemics which peak higher and faster. The peak prevalence and the attack rate, or final size of an unmitigated epidemic, are both determined by the R_0_, with larger R_0_ values giving rise to higher epidemic peaks and larger final sizes. Warmer temperatures have been shown to reduce the EIP and the generation time of arboviruses and to increase the vectorial capacity of Aedes populations and the R_0_ [49,53,82]. Taken together, these observations imply that climate change can enhance the probability of explosive outbreaks (Figure I), especially in locations that are currently unaffected by these diseases, once that competent vector populations establish, and arboviruses start circulating within the host population.

## Box 2 Traits and TPCs

Functional traits are measurable features or metrics of an organism that determine its fitness (ability to survive and reproduce). Thus, variation in functional traits can determine the abundance and fitness of populations of organisms. The effect of a specific trait on large-scale multi-species systems (such as those encountered in vector-borne disease biology) is often dynamic, nonlinear, and complex. This is primarily due to fundamental mechanistic links between a single trait such as body size, and many ecologically relevant processes such as feeding, predator evasion, and locomotion. For ectotherms, these functional traits are notably sensitive to environmental factors like temperature and humidity. The responses of these traits to environmental variables are generally nonlinear, adding extra complexity to already-complex systems. For temperature, trait responses are described by TPCs that tend to follow a specific functional form: a unimodal response, with a single peak at an intermediate temperature (T_opt_) that drops to 0 at extremes of high and low temperature (T_min_ and T_max_). Typically, probabilities (e.g., survival) are modelled using symmetric functions such as the quadratic function, and rates (i.e., traits per time) are modelled using asymmetrical functions such as the Brière function [53]. Both functions are defined in terms of the temperature T, the temperature extremes (T_min_ and T_max_) at which the trait hits zero and involve an extra parameter (c) characterising the overall shape of the curve, including the height of the peak. When these TPCs are used in place of a constant parameter in an equation, the equation becomes temperature-dependent in a natural manner. The traits of arbovirus vectors are typically split into three categories: inherent transmission traits, governing virus–vector interactions (e.g., the EIP and the transmission probability); life history traits, controlling mosquito population dynamics (e.g., survival and development rates); and external transmission traits, impacting the interactions and contact rates between vector populations and hosts (e.g., biting rates). Many of these traits often go unrecorded during empirical data collection, and even powerful imputation techniques such as multivariate imputation by chained equations [89] and Bayesian hierarchical probabilistic matrix factorization [90] cannot fill the current lack of data on some important vector traits.

## Clinician’s corner

The geographical expansion of arboviral diseases into new regions will pose new challenges to health systems and clinical practice in locations where they will emerge in the future.

Whilst most arbovirus infections are asymptomatic, mild and often self-limiting, the typically non-specific acute febrile symptoms developed during the early phases of arbovirus infections will pose challenges to the early clinical diagnosis of these diseases. In the absence of licensed prophylactic and therapeutic treatment, supportive fluid therapy is the main tool to manage plasma leakage, which is the primary cause of death. The current lack of sensitive and specific biomarkers identifying increased risks of developing severe disease during the early febrile phases of the illness will pose challenges to the appropriate management and treatment of haemorrhagic fevers in non-endemic settings, where education and training of medical staff will be required to target the treatment of these conditions to local populations. This is critical especially when these will occur in combination with comorbidities and chronic conditions (e.g., diabetes, obesity and heart diseases) that can exacerbate the severity of arbovirus diseases.

In endemic settings, changes in the age-structure of the population and specifically demographic shifts towards older populations will cause infections to occur later in life, thus coinciding with higher rates of diabetes, cardiovascular disease, hypertension, and obesity which can lead of increased severity and hospitalisation. Therefore, the continuous monitoring, adaptation and roll out of training activities will be needed also in endemic settings, to adequately respond to the changing demographics and health conditions of the affected patients.

The development of accurate and affordable rapid diagnostic tests – including point-of-care tests – could promote early diagnosis and outbreak detection, and enhance disease surveillance in both epidemic and endemic settings. Whilst several efforts have been made to identify biomarkers of severity, and correlates of severe disease (as well as protection), these are still lacking for several arboviruses. The establishment of correlates of protection and severe disease is a priority not only for clinical practice but also for the future development of vaccines and therapeutics.

## Outstanding questions

What is the current infection and disease burden of arboviruses across Africa?

Does the severity of arboviral diseases differ substantially between geographies, and if so, what drives the observed variation?

Are there fundamental differences between the microclimate experienced by vectors and the global gridded climate estimates used by most models?

How does rainfall affect the carrying capacity of different environments and the risk of disease transmission across geographies?

How do genetic variations in vector distribution, vectorial capacity, and thermal performance affect the current and future risk of arbovirus transmission?

How will climate change affect migration and mobility patterns, and how will these modify the risk of arbovirus transmission?

As humans move closer to animal populations, what is the increase in the spillover risk of sylvatic arboviruses?

How will climate change affect the ecology and behaviour of vectors and animal reservoirs, and what are the implications of these changes for disease transmission and control?

How will increasing temperatures modify the effect of vector-control strategies, including traditional insecticide-based methods and interventions based on the release of *Wolbachia*-carrying or genetically modified mosquitoes?
